# Could Fecal Microbiota Be a Useful Indicator of Serum Cholesterol Levels among Men?

**DOI:** 10.3390/jpm10040175

**Published:** 2020-10-16

**Authors:** HuiJun Chih, Stuart C. Smith, Ramon S. Hall, Stuart K. Johnson

**Affiliations:** 1 School of Public Health, Curtin University, Bentley, WA 6102, Australia; 2 School of Exercise and Nutrition Sciences, Deakin University, Burwood, VIC 3216, Australia; s_csmith@bigpond.net.au (S.C.S.); r.hall@deakin.edu.au (R.S.H.); 3 School of Molecular and Life Sciences, Curtin University, Bentley, WA 6102, Australia; s.johnson@curtin.edu.au

**Keywords:** gastrointestinal microbiota, health-promoting microbes, probiotics, cardiovascular risk factors, personalized healthcare, non-invasive diagnostics

## Abstract

Trepidation with blood tests among men may result in fewer routine screening and examination of their cardiovascular risk factors. Associations between fecal microbiota and serum cholesterols have not been well-established. The aim of this study was to explore such association in order to determine the potential of fecal microbiota as a non-invasive alternate predictor of serum cholesterols. Secondary data from a cross-over trial were analyzed. Associations between fecal microbiota, mainly *Bifidobacterium* and *Clostridial* group, of healthy men (*n* = 16) and their total cholesterols, low and high-density lipoprotein cholesterols (LDL-C and HDL-C) were assessed using generalized estimating equations, adjusted for diet intervention, diet order, frequency of defecation and flatulence level. For every two-fold increase in fecal *Bifidobacterium*, geometric mean of LDL-C increases by a factor of 1.23 (95% CI: 1.01, 1.49) whilst that of HDL-C increases by a factor of 1.07 (95% CI: 1.03, 1.10). For every two-fold increase in *Clostridial* group (*C. ramosum*, *C. spiroforme* and *C. cocleatum*), geometric mean of HDL-C decreases by a factor of 1.10 (95% CI: −1.16, −1.03). No association was found between total bacteria and serum cholesterols. Fecal *Bifidobacterium* spp. and *Clostridium* spp., are potential non-invasive surrogate markers of men’s serum cholesterols.

## 1. Introduction

Frequent screening, such as blood tests, may facilitate management of risk factors for cardiovascular diseases, but men are less likely to participate in screening services than women [1,2,3]. While the screening procedure can influence screening behavior, individual factors such as fear of pain was unexpectedly reported as one of the barriers to health screening among men [4]. Such trepidation can result in reluctance to undertake routine screening and/or examination attendance which results in blood taking among men, thereby increasing the mortality from cardiovascular diseases when men often underestimated their risk [4].

Studies investigated the health benefits of dietary fiber on serum lipid levels [5,6] that may affect risks of cardiovascular diseases albeit conclusions were inconclusive. There are also reports on the effect of lupin fiber on fecal microbiota [7] and the effect of microbiota on markers of metabolic disorders such as obesity and cardiovascular diseases [6]. The concept of fecal microbiota and serum lipid levels had not been considered in comparison with prebiotics and serum lipid levels. Acknowledging the current gap of knowledge, this study aimed to determine the association between fecal microbiota (specifically *Bifidobacterium* spp. and *Clostridial* group) and serum cholesterol levels (total cholesterol, low-density lipoprotein cholesterol (LDL-C) and high-density lipoprotein cholesterol (HDL-C) using data from a cross-over fiber-enriched trial. As this study aimed to investigate the potential of using fecal samples (instead of blood samples drawn through invasive procedures) to predict serum cholesterol levels, the finding may provide insight regarding the potential of fecal analysis as a non-invasive alternate indicator of serum lipid levels, which may further inform personalized healthcare.

## 2. Materials and Methods

### 2.1. Data Source

Secondary data were obtained from a single-blind, randomized, cross-over, 12-week dietary intervention examining the effect of high fiber diet on healthy middle-aged men who were slightly overweight with an acceptable healthy range of serum cholesterol levels [5]. Characteristics of the participants were reported in detail elsewhere [5]. Data of fecal microbiota (the predictors) and serum cholesterol levels (the outcome variables) along with other covariates were retrieved from 16 participants who participated in the trial.

Briefly, prior to the trial, the participants’ habitual energy intake over a 4-day period was measured to design individual energy density-matched experimental diets. During the trial, two groups of participants consumed a prescribed control diet and a prescribed fiber-enriched diet (17–30 g more than the control diet) per day, for 28 days followed by a wash-out period of 28 days, and consumed the alternate diet for 28 days [5]. The prescribed diets (manufactured by George Weston Foods in Australia) were equivalent in macronutrient composition and fatty acid compositions, except for dietary fiber. The control diet contained a daily amount of 25 g of fiber and the fiber-enriched diet contained a daily amount of 55 g of fiber for those who consumed a total energy intake of greater than 9 MJ/day (or 18 g and 35 g dietary fiber per day, respectively, for total energy intake of 9 MJ/day or less) [5]. The fiber was derived from the thickened cell walls of the seeds of the legume lupin (*Lupinus angustfolius*) and displaced some of the available carbohydrates in the manufactured foods [5]. The study was approved by the Deakin University Ethics Committee.

#### 2.1.1. Predictors

Fecal total bacteria, *Bifidobacterium*, and the *Clostridial* group involving *C. ramosum*, *C. spiroforme,* and *C. cocleatum*, were measured using a fluorescent in situ hybridisation (FISH) assay protocol [7]. Briefly, aliquots (0.5 g) of the fecal sample were homogenized and cells of the fecal debris were fixed in 3% paraformaldehyde overnight before being diluted and applied to gelatin-coated slides and fixed to slides with 96% ethanol and hybridized with oligonucleotide probes [7]. The probes used in the FISH assay were ‘gold standard’ oligonucleotide probes [7,8]. The fluorescent-labelled probes used were the Bact338 probe, the Bif164 probe, and the Cspiro 222 probe for total bacteria, genus *Bifidobacteria*, and the group of *Clostridium ramosum*, *C. spiroforme,* and *C. cocleatum*, respectively [7]. Contamination of samples from other microbiota was minimized due to the procedure of FISH sample preparation (collection in sterile container, paraformaldehyde fixation of fecal samples inhibiting microbial growth, and the conditions of hybridization using specific oligonucleotide probes which recognize specific bacteria only). The amount of bacteria present in the sample was reported as colony-forming units (CFU/g dry weight) by correcting number of cells/g wet weight by measuring water content.

Whilst pyrosequencing is more recent in identifying fecal microbiota, group-specific probes by FISH analyses can identify 80% of the total bacteria [8] and can therefore identify significant changes in the bacteria. The levels of *Bifidobacterium* spp. and *Clostridial* group were the focus of this current study because they are the gut-surface associated microbiota and based upon the FISH analyses undertaken in the previous study [7] showing that levels of *Bifidobacterium* spp are promoted in the gut with increased intake of dietary fiber whilst pathogenic bacteria such as the *Clostridial* group are significantly reduced [7]. Together these microbiota could serve as good predictors of the risk of inflammation or metabolic disorders, such as a change in serum cholesterol levels.

#### 2.1.2. Outcome Variables

Serum total cholesterol level, LDL-C and HDL-C were measured using enzymatic colorimetric methods and LDL-C was calculated using Friedewald’s equation as detailed elsewhere [5].

#### 2.1.3. Covariates

Frequency of defecation was measured using a self-reported line scale with a left-hand anchor ‘far less often than usual’, a mid-point anchor ‘same as usual’, and a right-hand anchor ‘much more often than usual’ [9]. Participants also self-reported their flatulence level on a line scale with a left-hand anchor ‘far less than usual’ a mid-point anchor ‘same as usual’, and a right-hand anchor ‘much more than usual’ [9].

### 2.2. Statistical Analyses

The normality of the predictors (fecal microbiota) and outcome variables (serum cholesterols) were assessed using skewness coefficient and visualization of a histogram and normal probability plot. Since the microbiota were skewed, descriptive statistics of microbiota were reported in median and interquartile range whilst descriptive statistics of the normally distributed serum cholesterol levels were reported in mean and standard deviation. 

Generalized estimating equations (GEE) method [10] was applied to estimate the associations between microbiota (the predictors) and serum cholesterol levels (the outcome variables) because participants were nested within groups in the cross-over trial and hence observations between the participants were clustered [10]. The GEE method is appropriate to assess associations of clustered data [10]. Based on previously published literature [9], covariates including diet intervention and order, frequency of defecation, and flatulence level were controlled for in the unstructured GEE models with fecal microbiota as the predictors and serum cholesterol levels as the outcome variables. The coefficients generated from the GEE models corresponding to each predictor can be used to calculate the serum cholesterol levels, thus facilitating the investigation of the potential of fecal analysis as a non-invasive alternate indicator of serum lipid levels. In order to facilitate interpretation of the association between log-transformed microbiota data and serum cholesterol levels, geometric means were calculated based on the coefficients. The significance level was set at 0.05. All analyses were completed using Stata 12.0 (StataCorp, College Station, TX, USA).

## 3. Results

The median colony forming units (CFU/g dry weight) of total bacteria and *Bifidobacterium* among participants who had the fiber-enriched diet first appeared to be higher than those reported by the same participants who had the control diet first (Table 1). Nevertheless, the *C. ramosum*, *C. spiroforme,* and *C. cocleatum* group levels were similar for both dietary interventions independent of diet order (Table 1). Similarly, the mean total cholesterol, LDL-C and HDL-C levels were comparable for both diet interventions, at around five, three, and one mmol/L (Table 1).

As shown in Table 2, there is a strong association between levels of *Bifidobacterium* and both low-density lipoprotein cholesterol (LDL-C) and high-density lipoprotein cholesterol (HDL-C) but not serum total cholesterol level. After controlling for diet intervention and order, frequency of defecation, and flatulence level in the GEE model, it was found that for every two-fold increase in fecal *Bifidobacterium* level, the geometric mean of low-density lipoprotein cholesterol (LDL-C) increases by a factor of 1.23 (95% CI: 1.01, 1.49) (Table 2). Similarly, there is a significant increase in HDL-C by a factor of 1.07 (95% CI: 1.03, 1.10). However, an increase in fecal *Bifidobacterium* level was not found to be associated with any significant change in serum total cholesterol level (Table 2).

There is also a strong association between levels of the *Clostridial* group (*C. ramosum*, *C. spiroforme,* and *C. cocleatum*) and HDL-C but not with serum total cholesterol nor LDL-C (Table 2). After controlling for the covariates, for every two-fold increase in fecal *C. ramosum*, *C. spiroforme,* and *C. cocleatum* group level, the geometric means of HDL-C reduces by a factor of 1.10 (95% CI: −1.16, −1.03) (Table 2). However, there is no strong evidence for an association between levels of *C. ramosum*, *C. spiroforme,* and *C. cocleatum* and serum total cholesterol levels or LDL-C (Table 2).

Whilst the total bacteria probe (Bac 388 probe) could measure about 80% of total bacteria [9] there was no significant evidence for an association between levels of total bacteria and total cholesterol (Table 2). Similarly, no significant association was found between levels of total bacteria and LDL-C or HDL-C levels (Table 2).

## 4. Discussion

Previous findings report the effect of diet on changes in microbiota [7], and the effect of diet on changes in serum cholesterols [5,11]. However, a relationship between the microbiota and serum cholesterol levels irrespective of diet has not been studied comprehensively. Through investigating the association between fecal microbiota and serum cholesterol levels using data from a cross-over randomized trial (level I evidence) and keeping the effect of diet constant in the model, the present study highlighted the novel potential of fecal analysis (and specific bacteria) as a non-invasive alternate indicator of serum lipid levels in men.

Gut microbiota are involved in nutrient metabolism and influence different metabolites (such as cholesterol and other dietary components) being absorbed, stored, or excreted in the fecal matter [6,11]. Whilst bacteria within the gut microbiota, especially the probiotic bacteria, have bile salt hydrolase that may lower cholesterol, this activity depends on the interaction between the bile salt hydrolase and the host, and the mechanism of *Bifidobacterium* spp. on human cholesterol levels is less understood due to the inconclusive evidence [11]. Despite *Clostridium* playing a key role in gut homeostasis [12], the negative association between a major *Clostridial* group (*C. ramosum*, *C. spiroforme,* and *C. cocleatum*) and HDL-C (as opposed to the positive association between *Bifidobacterium* spp. and HDL-C) determined in the present study is novel. To the best of the authors’ knowledge, there is no comparable study investigating such a relationship to enable in-depth discussion of the findings.

Despite following a rigorous study design, this study has several limitations: (1) The homogenous sample of Caucasian healthy men reduces the ability to extrapolate these result validly to men of other ages and ethnicity; (2) The diet was restricted to a lupin fiber-enriched diet which limited assessment of the association between microbiota generated from protein-enriched or other diets, including other fiber-enriched diets and serum cholesterol levels; (3) The spectrum of microbiota studied may not be fully extensive and other microbiotas were not specifically measured. Despite these limitations, a relationship was shown to exist between the *Bifidobacterium* spp. and serum cholesterol levels (LDL-C and HDL-C) as well as between a *Clostridial* group (*C. ramosum*, *C. spiroforme,* and *C. cocleatum*) and HDL-C. Future studies may confirm the potential of fecal matter microbiota as an alternate non-invasive surrogate markers of serum LDL-C and HDL-C (and potentially the risk profile of cardiovascular diseases) using data from other dietary cross-over trials. Trials that recruited men (and women) of various ages and ethnic groups with or without other comorbidities who ingested different food groups known to affect the structure and metabolome of the intestinal microbiota contributing to disorders such as cardiovascular diseases would contribute key information regarding the potential of fecal microbiota as an alternate non-invasive biomarker. The findings may remove barriers associated with trepidation of pain during an individual’s (especially men’s) health examination, thereby increasing uptake of health screening and reducing preventable deaths from cardiovascular diseases.

Another application of this novel finding could be encouraging to men as an add-on analysis of fecal samples collected as part of the national bowel cancer screening program to identify people at risk of developing cardiovascular diseases and correlate individual serum LDL levels with their microbiota profile at the same time. This approach may assist routine health diagnostics rather than initiating a separate program that may incur unnecessary logistics costs. Future studies may also include analyses of the costs of stool analysis (in comparison to blood plasma diagnostics) and replacing medications by lifestyle (diet or fiber intake) while considering types of health insurance policies that may cover for these costs in different countries.

## 5. Conclusions

Fecal matter (especially using the *Bifidobacterium* level) has the potential to act as a general indicator of serum LDL-C and HDL-C levels in men. It is imperative that future larger studies generate more data to confirm such effect to allow the development of algorithm-based models to estimate cholesterol levels without the need for invasive blood tests.

## Figures and Tables

**Table 1:** Descriptive statistics of fecal microbiota and serum cholesterol levels reported as either median (interquartile range) or mean ± standard deviation.

	Faecal Microbiota (CFU/g Dry Weight)			Serum Cholesterol Levels (mmol/L)		
	*Bifidobacterium*	*C. ramosum, C. spiroforme and C. cocleatum group*	Total bacteria	Total cholesterol	Low-density lipoprotein (LDL-C)	High-density lipoprotein (HDL-C)
^1^ Diet intervention order 1	1.01 (2.18) × 10^9^	0.05 (0.03) × 10^9^	45.9 (43.0) × 10^9^	4.86 ± 0.92	3.00 ± 0.85	1.27 ± 0.29
^1^ Diet intervention order 2	0.37 (0.59) × 10^9^	0.07 (0.04) × 10^9^	36.6 (53.8) × 10^9^	5.34 ± 1.00	3.53 ± 0.94	1.27 ± 0.32

^1^ Diet intervention order 1: fiber-enriched diet first then control diet; Diet intervention order 2: control diet first then fiber-enriched diet.

**Table 2:** Associations between every two-fold increase in the log-transformed fecal microbiota and geometric means ^1^ (95% confidence intervals) of men’s serum total cholesterol, low density lipoprotein (LDL-C), high density lipoprotein (HDL-C) levels.

Fecal Microbiota	Total Cholesterol	LDL-C	HDL-C
*Bifidobacterium*	1.13 (−1.07, 1.37)	**1.23** **(1.01, 1.49)**	**1.07** **(1.03, 1.10)**
*C. ramosum, C. spiroforme* and *C. cocleatum*	1.34 (−2.04, 1.13)	1.14 (−1.72, 1.32)	**−** **1.10** **(−** **1.16, −** **1.03)**
Total bacteria	-1.31 (−1.72, 1.01)	2.34 (−1.60, 1.08)	1.04 (−1.00, 1.09)

^1^ Geometric means of serum cholesterol levels (mmol/L) were calculated based on coefficients β* of log-transformed microbiota data (CFU/g dry weight) generated from the generalized estimating equations method from clustered data obtained from a cross-over randomized controlled trial, adjusted for diet intervention and order, frequency of defecation and flatulence level. Positive values indicate an increase in serum cholesterol levels while negative values indicate a decrease in serum cholesterol levels for every 2-fold increase in the microbiota levels. Numbers presented in bold indicate a significant association between the log-transformed microbiota and serum cholesterol levels (*p* < 0.05).
